# Laparoscopic common bile duct exploration in patients with previous abdominal biliary tract operations

**DOI:** 10.1007/s00464-020-07429-3

**Published:** 2020-02-18

**Authors:** Min Li, Ying Tao, Sheng Shen, Lujun Song, Tao Suo, Han Liu, Yueqi Wang, Dexiang Zhang, Xiaoling Ni, Houbao Liu

**Affiliations:** 1 Department of General Surgery, Zhongshan Hospital, Fudan University, 180, Fenglin Road, Shanghai, 200032 China; 2 Department of General Surgery, Xuhui Central Hospital, Shanghai, 200031 China

**Keywords:** Common bile duct, Laparoscopic, Choledocholithiasis, Abdominal adhesions, Biliary tract surgical procedures, Postoperative complications

## Abstract

**Background:**

A history of abdominal biliary tract surgery has been identified as a relative contraindication for laparoscopic common bile duct exploration (LCBDE), and there are very few reports about laparoscopic procedures in patients with a history of abdominal biliary tract surgery.

**Methods:**

We retrospectively reviewed the clinical outcomes of 227 consecutive patients with previous abdominal biliary tract operations at our institution between December 2013 and June 2019. A total of 110 consecutive patients underwent LCBDE, and 117 consecutive patients underwent open common bile duct exploration (OCBDE). Patient demographics and perioperative variables were compared between the two groups.

**Results:**

The LCBDE group performed significantly better than the OCBDE group with respect to estimated blood loss [30 (5–700) vs. 50 (10–1800) ml; *p* = 0.041], remnant common bile duct (CBD) stones (17 vs. 28%; *p* = 0.050), postoperative hospital stay [7 (3–78) vs. 8.5 (4.5–74) days; *p* = 0.041], and time to oral intake [2.5 (1–7) vs. 3 (2–24) days; *p* = 0.015]. There were no significant differences in the operation time [170 (60–480) vs. 180 (41–330) minutes; *p* = 0.067]. A total of 19 patients (17%) in the LCBDE group were converted to open surgery. According to Clavien’s classification of complications, the LCBDE group had significantly fewer postoperative complications than the OCBDE group (40 vs. 57; *p* = 0.045). There was no mortality in either group. Multiple previous operations (≥ 2 times), a history of open surgery, and previous biliary tract surgery (including bile duct or gallbladder + bile duct other than cholecystectomy alone) were risk factors for postoperative adhesion (*p* = 0.000, *p* = 0.000, and *p* = 0.000, respectively).

**Conclusion:**

LCBDE is ultimately the least invasive, safest, and the most effective treatment option for patients with previous abdominal biliary tract operations and is especially suitable for those with a history of cholecystectomy, few previous operations (< 2 times), or a history of laparoscopic surgery.

According to research results from different centers around the world, common bile duct (CBD) stones are detected in approximately 5 to 21% of patients undergoing surgery for symptomatic gallbladder stones [1]. CBD stones can result in biliary colic, obstructive jaundice, hepatic abscess, acute cholangitis, and pancreatitis. Traditional surgical treatment comprises intraoperative cholangiography to detect the presence of bile duct calculi followed by choledocholithotomy and T-tube placement [2]. Currently, endoscopic retrograde cholangiopancreatography (ERCP) and laparoscopic CBD exploration (LCBDE) are the most well-known, minimally invasive treatment options [3]. ERCP has remained the preferred invasive approach for managing symptomatic CBD stones for decades. However, ERCP always requires a two-stage approach (ERCP before or after laparoscopic cholecystectomy (LC)), although an increasing number of clinical centers try to perform ERCP during an operation. In addition, ERCP can sometimes cause serious ERCP-related complications, such as bleeding, duodenal perforation, cholangitis, and pancreatitis. Moreover, it may be difficult or impossible to clear large, multiple, intrahepatic ducts or impacted stones in the CBD by ERCP, and patients with these symptoms can benefit from open CBD exploration (OCBDE) or LCBDE, as these procedures have a high success rate in salvaging such stones [4]. In addition, it has been reported that endoscopic sphincterotomy (EST) may cause duodenobiliary, pancreatic juice and intestinal content reflux that results in recurrent bile duct stone formation, cholecystitis, inflammation in the bile duct, and cholangiocarcinoma [5]. Currently, some experts also suggest that ERCP be applied with appropriate indications in clinical practice.

For decades, laparoscopic skills have undergone considerable development. Since LCBDE was first introduced in 1991, it has been proven to be a safe, reliable, and effective method for CBD stones and has gained widespread acceptance with its additional advantage of being a single-stage approach [3]. Recent meta-analyses have found LCBDE to be superior to a two-stage approach (ERCP before or after LC) with regards to the total length of hospital stay, CBD stone clearance rate, and medical costs [6]. Additionally, LCBDE has the advantages of preserving the function of the sphincter of Oddi (SO), with lower morbidity and fewer ERCP-related complications [7, 8]. However, a history of prior abdominal operations posed a significant challenge for LCBDE because of the surgically altered gastrointestinal anatomy and adhesions [9].

Nevertheless, with the development of laparoscopic techniques and technological advances in equipment, a growing number of reports have suggested that laparoscopic procedures can also be performed as a standard treatment in patients with a history of abdominal operations [10, 11]. However, there are very few reports about laparoscopic procedures in patients with a history of abdominal biliary tract surgery. Thus, the objective of this study was to assess the effectiveness of LCBDE and evaluate the factors affecting the degree of postoperative adhesion in patients with previous abdominal biliary tract surgery. In the present study, we assessed the feasibility, safety, and influencing factors of postoperative adhesion of LCBDE in patients with CBD stones after abdominal biliary tract surgery and compared these factors with those of open surgery.

## Methods

### Patients and grouping

A total of 227 consecutive patients with CBD stones after abdominal biliary tract surgery who underwent surgical treatment at Zhongshan Hospital, Fudan University between December 2013 and June 2019 were retrospectively analyzed. Patients were grouped according to the surgical options: those who underwent LCBDE (*n* = 110) and those who underwent OCBDE (*n* = 117).

The diagnosis of CBD stones was based on conventional imaging tests, such as routine abdominal ultrasound, magnetic resonance cholangiopancreatography (MRCP) and relevant laboratory tests, including a routine blood examination, liver function tests (LFTs), and blood coagulation function tests. Medical clinical data were reviewed retrospectively, and the operative outcomes (including operative time, estimated blood loss, remnant CBD stones, open conversion, and postoperative hospital stay) and postoperative complications (including pulmonary diseases, bile leakage, wound infection, and postoperative bleeding) were collected and analyzed. The study protocol was approved by the institutional ethical committees of Zhongshan Hospital.

### Definitions

Postoperative adhesions were divided into four classes according to the Operative Laparoscopy Study Group (OLSG) classification and adhesion scoring system proposed by Luciano et al. as follows: 0—no adhesions; 1—filmy, avascular adhesions and easily separable; 2—dense and/or vascular adhesions; and 3—cohesive adhesions [12, 13]. A wound infection involves only the skin or subcutaneous tissue and requires the documentation of one or more of the following: purulent drainage from the wound, aseptically obtained wound culture with isolated organisms, opening of the wound by a physician with clinical symptoms of infection, or diagnosis by a physician [14]. Postoperative pulmonary complications in our study mainly included pleural effusion, atelectasis and pneumonia. Hypertension and diabetes mellitus were diagnosed and confirmed by a specialist on the basis of clinical diagnostic criteria. Cardiovascular disease in our study mainly included coronary artery diseases, cardiomyopathy, abnormal heart rhythms, and myocardial ischemia. Pulmonary disease in our study mainly included asthma, chronic obstructive pulmonary disease, pulmonary fibrosis and pneumonia. Liver disease in our study mainly included viral hepatitis and cirrhosis. Cerebral diseases in our study mainly included cerebral infarction, cerebral thrombosis and cerebral hemorrhage.

### Inclusion and exclusion criteria

The inclusion criteria were as follows: (1) a diagnosis of CBD stones confirmed by ultrasound, computerized tomography or MRCP before surgery, (2) patients who were not suitable for ERCP (e.g., those with stone relevant factors (multiple, large, impacted, and hilar stones), those with anatomic changes from the duodenal papilla, those who underwent some kind of gastrointestinal surgery, and those who could not tolerate or cooperate with or were hypersensitive to iodinated contrast media), (3) patients who failed ERCP in our institution or failed cannulation or those with incomplete stone clearance, and (4) patients with a history of at least one abdominal biliary tract surgery including LCBDE and OCBDE.

The exclusion criteria were as follows: (1) patients who did not have a history of abdominal biliary surgery and (2) patients who had contraindications for laparoscopic or open surgery.

### Surgical procedure

All the procedures (including laparotomy and laparoscopic surgery) were performed by an experienced surgeon.

### LCBDE technique

Patients in the LCBDE group were placed in a reverse Trendelenburg position under general anesthesia. We tended to use a three-trocar method: the first 10 mm trocar was placed in the subumbilical area for carbon dioxide insufflation and a 30° angled laparoscope. The second 10 mm trocar was located in the area below the xiphoid. The 5 mm and third trocar was placed in the midclavicular line, 1–2 cm under the right costal margin. Sometimes, an additional 5 mm trocar in the right anterior axillary line was needed when the adhesion was serious in the operation area. Cholecystectomy was performed first unless the patient had previously undergone the procedure. Careful dissection was used to identify the CBD, where a choledochotomy was performed with an approximately 1–1.5 cm longitudinal incision on the anterior surface. Then, a 5 mm flexible choledochoscope was regularly applied to find and remove stones with a stone basket, saline flushing, or electrohydraulic lithotripsy.

After clearance of the stones, a choledochoscope was used to confirm whether stones remained in the intrahepatic duct and CBD. If there was no residual stone, the choledochotomy incision was closed with a Vicryl 4–0 interrupted suture. For patients with a residual stone, a latex T-tube was placed at the operating surgeon’s discretion according to the condition of the CBD, which was then sewn with the same suture. A T-tube cholangiogram was performed 2–4 weeks postoperatively for patients who had T-tube drainage, and the T-tube was removed if there was no residual stone in the biliary system. For patients with a history of abdominal biliary tract surgery, the site of the first trocar was often chosen far from the original surgical site.

### Open surgery technique

Patients in the open surgery group were placed in a reverse Trendelenburg position under general anesthesia, and the surgery was performed via a right upper quadrant subcostal incision. First, abdominal adhesions were separated carefully with blunt/sharp dissection, electrocautery or a harmonic scalpel. Cholecystectomy was performed first unless the patient had previously undergone the procedure. The CBD was dissected, and a choledochotomy was performed with an approximately 1.5 cm longitudinal incision on the anterior surface of the CBD. Last, the choledochoscope was regularly applied, and a T-tube was placed if necessary.

### Postoperative treatment and follow-up

The patients were encouraged to be up and about as early as possible in the early stages after the surgery, and anticoagulant therapy could begin 24 h after surgery for patients at a high risk of thrombosis. Routine blood and blood biochemical examinations were performed every 72 h. An abdominal ultrasound was performed during the postoperative monitoring of fluid accumulation in the operative area for patients if necessary. If the patient had a cough, fever, or suspicious pleural effusion, chest computerized tomography was performed. If an abdominal cavity drainage tube was placed during the operation, it was removed when the drainage had stopped or became less than approximately 25 mL/day. If a T-tube was placed during the operation, it could be lifted up or clamped approximately one week after the patient was discharged. Outpatient follow-up was recommended for all patients 4 weeks after surgery, every 2 months for a minimum of 6 months, and imaging studies such as ultrasound or computed tomography were performed if there were problems. No patient was lost to follow-up.

### Statistics

Classification variables are expressed as absolute numbers and percentages, while continuous variables are expressed as medians and ranges. Data analysis was performed using SPSS 20.0 (IBM, USA). Classification variables were analyzed between groups using the chi-square test or Fisher’s exact test, as appropriate. Continuous variables were analyzed between groups using the *t* test. The Cochran–Armitage test for trend was used in the categorical data analysis, which was performed with SAS 9.13 (Statistical Analysis System, USA). *p* < 0.05 was considered statistically significant.

## Results

### Patient characteristics

Between December 2013 and June 2019, a total of 227 patients who met the inclusion criteria were included in this study. A total of 110 patients underwent LCBDE, and 117 patients underwent OCBDE. Demographic details and relevant preoperative examination results are shown in Table 1. The results showed no obvious differences in terms of comorbidities such as hypertension, cardiovascular disease, pulmonary disease, liver disease, or cerebral diseases between the two groups, but there were more patients with diabetes mellitus in the LCBDE group than in the OCBDE group (*p* = 0.017). Moreover, both groups had similar histories of biliary surgery and American Society of Anesthesiologists (ASA) scores (*p* = 0.796 and *p* = 1.557, respectively). In addition, there were no obvious differences regarding the CBD size between the two groups (*p* = 0.705). However, there was a significant difference in the preoperative LFTs between the LCBDE group and the OCBDE group.

**Table 1:** Demographics and preoperative data of 227 patients included in the current study

Parameter	LCBDE (*n* = 110)	OCBDE (*n* = 117)	*p* value
Sex, *n* (%)			0.780
Male	45 (41)	50 (43)	
Female	65 (59)	67 (57)	
Age, years (range)	65 (34–90)	63 (23–90)	0.166
BMI, (range)	20.42 (18.19–28.03)	20.07 (18.61–25.60)	0.152
Comorbidity (%)			
Hypertension	36 (33)	30 (26)	0.240
Diabetes mellitus	24 (22)	12 (10)	0.017
Cardiovascular disease	11 (10)	8 (7)	0.390
Pulmonary disease	1 (1)	4 (3)	0.404
Liver disease	4 (4)	0 (0)	0.115
Cerebral diseases	7 (6)	3 (3)	0.284
Others	7 (6)	8 (7)	0.886
Biliary surgery history, *n* (%)			0.796
Cholecystectomy	66 (60)	66 (56)	
Choledochotomy	11 (10)	11 (9)	
Cholecystectomy + choledochotomy	33 (30)	40 (34)	
ASA score, *n* (%)			1.557
I, II	106 (96)	107 (91)	
III, IV, V	4 (4)	10 (9)	
Preoperative LFTs and radiologic findings			
Alkaline phosphatase (U/L)	215 (161–270)	125 (47–854)	0.005
Alanine aminotransferase (U/L)	23.3 (4–394)	37.8 (5–910.3)	0.003
Aspartate aminotransferase (U/L)	24.1 (6.2–421.4)	35 (10.9–1586.9)	0.008
Bilirubin (mg/dL)	14 (4.1–200.8)	16.0 (5.2–269.8)	0.001
CBD size in ultrasound (mm)	12 (4–30)	11.5 (4–35)	0.705

Values are presented as the *n* (%) or median (range)

*CBD* common bile duct, *dL* deciliter, *LCBDE* laparoscopic common bile duct exploration, *mg* milligram, *mm* millimeter, *OCBDE* open common bile duct exploration, *LFTs* liver function tests

### Operative outcomes and postoperative recovery

The operative outcomes are presented in Table 2. There was no significant difference in terms of the mean operation time between the LCBDE group and the OCBDE group (*p* = 0.067). The LCBDE group performed better than the OCBDE group with respect to estimated blood loss, remnant CBD stones, postoperative hospital stay and time to oral intake, with significant differences between the two groups (*p* = 0.041, *p* = 0.050, *p* = 0.041, and *p* = 0.015, respectively). A total of 19 patients (17%) in the LCBDE group were converted to open surgery. The reasons for open conversion were extensive intra-abdominal adhesions or severe fibrosis at the porta hepatis in 14 patients, incisional hernia near the original incision in 2 patients, a large number of bile duct stones around the hepatic hilar region in 1 patient, impacted stones in the left intrahepatic bile duct in 1 patient, and serious cirrhosis with varicose vessels on the surface of the CBD in 1 patient. With respect to postoperative complications, pulmonary diseases such as pneumonia and pleural effusion seemed to be the most common complications, affecting up to 32% and 42% of the LCBDE and OCBDE groups, respectively. In addition, bile leakage was the second most common postoperative complication, affecting 6% and 10% of the LCBDE and OCBDE groups, respectively. The patients with bile leakage recovered through symptomatic and supportive treatment, such as local drainage and anti-infective therapy. Empiric antibiotic treatment was first given for patients with infection, and the antibiotics were sometimes adjusted according to bacterial culture and drug sensitivity tests, especially for patients with severe infections. Moreover, wound infection was the third most common postoperative complication, affecting 3% and 3% of the LCBDE and OCBDE groups, respectively. The patients with wound infections recovered through wound dressing and local drainage. Finally, postoperative bleeding was found only in 3 patients. Exploratory laparotomy and digital subtraction angiography were applied to 1 patient in the LCBDE group, and the hemorrhage was ultimately found in a branch of the superior pancreaticoduodenal artery, which was treated by means of intravascular embolization. Digital subtraction angiography was applied to 1 patient in the OCBDE group, and the hemorrhage was ultimately found in a branch of the left hepatic artery, which was treated by means of intravascular embolization. An exploratory laparotomy was applied to another patient in the OCBDE group, and the hemorrhage was found in the gallbladder bed. Most importantly, there were no obvious differences in terms of the prevalence of pulmonary diseases (*p* = 0.117), bile leakage (*p* = 0.102), wound infection (*p* = 1.000), or postoperative bleeding (*p* = 1.000) between the two groups. There was no mortality in either group at the end of follow-up.

**Table 2:** Comparison of operative outcomes between groups

Parameter	LCBDE (*n* = 110)	OCBDE (*n* = 117)	*p* value
Operative time (min)	170 (60–480)	180 (41–330)	0.067
Estimated blood loss (mL)	30 (5–700)	50 (10–1800)	0.041
Remnant CBD stones, *n* (%)	19 (17)	33 (28)	0.050
Open conversion, *n* (%)	19 (17)	–	–
Postoperative hospital stay (days)	7 (3–78)	8.5 (4.5–74)	0.041
Time to oral intake	2.5 (1–7)	3 (2–24)	0.015
Pulmonary diseases, *n* (%)	35 (32)	49 (42)	0.117
Bile leakage, *n* (%)	5 (5)	12 (10)	0.102
Wound infection, *n* (%)	3 (3)	3 (3)	1.000
Postoperative bleeding, *n* (%)	1 (1)	2 (2)	1.000
Others, *n* (%)	1 (1)	4 (3)	0.404
Mortality, *n* (%)	0 (0)	0 (0)	–

*min* minute, *mL* milliliter

### Clavien’s classification of complications

According to Clavien’s classification of complications, Clavien grades II and IV were observed only in the OCBDE group, and Clavien grade V was not observed in either group. Clavien’s classification of complications is shown in Table 3. First, the postoperative course was normal (Clavien grade I) in most patients, and Clavien grade I accounted for 34% of the LCBDE group and 39% of the OCBDE group. Second, Clavien grade II complications in the OCBDE group included pleural effusion in 2 patients, bile leakage in 2 patients, and gastroparesis in 1 patient. Third, there were 3 and 6 Clavien grade III complications in the LCBDE and OCBDE groups, respectively. In the LCBDE group, 1 patient with intra-abdominal bleeding received exploratory laparotomy and digital subtraction angiography as mentioned above; 1 patient with postoperative intra-abdominal infection received ultrasound-guided drainage of abdominal fluid and anti-infective treatment; 1 patient with postoperative arytenoid dislocation received postoperative restoration treatment. In the OCBDE group, 2 patients with intra-abdominal bleeding received exploratory laparotomy and digital subtraction angiography separately, as mentioned above. Two patients with bile leakage and another 2 patients with pleural effusion recovered through ultrasound-guided local drainage. Finally, one patient with Clavien grade IV complications in the OCBDE group was diagnosed with acute renal failure after surgery and recovered after a series of treatments, including hemodialysis, anti-inflammatory drugs, and fluid replacement. Statistically significant differences in complications were discovered between the two groups (*p* = 0.045).

**Table 3:** Clavien’s classification of complications

Clavien’s grade	LCBDE (*n* = 110)	OCBDE (*n* = 117)	*p* value
I, *n* (%)	37 (34)	46 (39)	0.045
II, *n* (%)	0 (0)	5 (4)	
III, *n* (%)	3 (3)	6 (5)	
IV, *n* (%)	0 (0)	1 (1)	
V, *n* (%)	0 (0)	0 (0)	

### Factors affecting the degree of postoperative adhesion

Adhesions in the abdomen were seen mainly between the gallbladder liver bed and the neighboring organs (omentum, duodenum, right colon, and small bowel loops) or between the abdominal wall peritoneum [15]. Adhesions were divided into four classes according to the OLSG classification and adhesion scoring system proposed by Luciano et al. [12, 13, 15, 16]. The operation video was viewed by two operators and two reviewers. They independently scored each postoperative adhesion according to the OLSG classification. The overall adhesion score was 185 in the LCBDE group and 201 in the OCBDE group, and there was no significant difference between the two groups (*p* = 0.563). We further analyzed the factors affecting the degree of postoperative adhesion, which are shown in Table 4. In most patients, the postoperative adhesions were not serious; classes zero and 1, and class 2 accounted for 44% and 40% of all patients, respectively, while class 3 accounted for only 16% of all patients. There was no significant difference in sex, age, time of biliary disease (the duration of biliary disease from onset to admission), or time interval (the time interval between the first operation and the most recent operation) among classes zero and 1, 2 and 3 (*p* > 0.05). There was a significant difference in the previous number of operations, previous surgical approach and previous operation scope among classes zero and 1, 2 and 3 (*p ≤ *0.05). After further comparison between groups with respect to the previous operation scope, we found a significant difference between previous gallbladder surgery and previous bile duct surgery and previous gallbladder surgery and previous gallbladder + bile duct surgery between classes zero and 1, 2 and 3 (*p* = 0.000 and *p* = 0.000, respectively). However, there was no significant difference between previous bile duct surgery and previous gallbladder + bile duct surgery between classes zero and 1, 2 and 3 (*p* = 0.245). Therefore, the above results demonstrated that multiple previous operations (≥ 2 times), a history of open surgery, and previous biliary tract surgery (including bile duct or gallbladder + bile duct other than cholecystectomy alone) were risk factors for postoperative adhesion.

**Table 4:** Factors affecting the degree of postoperative adhesion

Parameter	All patients (*n* = 227)			*p* value
Adhesion type^a^	0 and 1	2	3	
Number, *n* (%)	99 (44)	91 (40)	37 (16)	
Sex, *n* (%)				0.093
Male	47 (49)	36 (38)	12 (13)	
Female	52 (39)	55 (42)	25 (19)	
Age, *n* (%)				0.365
≤ 60 years	40 (44)	31 (34)	20 (22)	
> 60 years	59 (43)	60 (44)	17 (13)	
Time of biliary disease^b^, *n* (%)				0.103
≤ 30 days	59 (47)	50 (40)	16 (13)	
> 30 days	40 (39)	41 (40)	21 (21)	
Previous number of operations, *n* (%)				0.000
1 time	95 (52)	64 (35)	22 (12)	
≥ 2 times	4 (9)	27 (59)	15 (33)	
Time interval^c^, *n* (%)				0.150
≤ 10 years	57 (50)	39 (34)	18 (16)	
> 10 years	42 (37)	52 (46)	19 (17)	
Previous surgical approach, *n* (%)				0.000
Open surgery	64 (36)	80 (45)	34 (19)	
Laparoscopic surgery	35 (71)	11 (22)	3 (6)	
Previous operation scope, *n* (%)				0.000
Gallbladder	86 (65)	35 (27)	11 (8)	
Bile duct	6 (27)	10 (45)	6 (27)	
Gallbladder + bile duct	7 (10)	46 (63)	20 (27)	

^a^According to the Operative Laparoscopy Study Group (OLSG) classification

^b^The duration of biliary disease from onset to admission

^c^The time interval between the first operation and the most recent operation

## Discussion

In recent decades, with the development of new laparoscopic techniques and devices, LC has been recognized as the gold standard for the surgical management of gallstone diseases [17]. At the same time, the treatment for CBD stones has changed, and an increasing number of options are now prevalent. These new options include ERCP, endoscopic papillary balloon dilation (EPBD), or EST (if necessary) followed by LC; LC and intraoperative transcystic or direct CBD exploration; postoperative ERCP with EPBD/EST and stone extraction; intraoperative ERCP, EPBD/EST, and stone extraction during LC; and conversion to laparotomy [18, 19]. Therefore, there are two main surgical strategies in clinical practice: a single-stage surgical strategy involving LCBDE and stone retrieval at the time of LC and an alternative option of staged procedures, with LC and ERCP on separate dates for CBD stone clearance [20].

The advantage of staged procedures is the significantly shorter surgical time, but patients are exposed to the risks associated with ERCP, such as infection, pancreatitis, hemorrhage, and perforation. In addition, delays between the two stages of treatment may result in prolonged hospital admission [20, 21]. Furthermore, both endoscopic papillary large balloon dilation (EPLBD) alone and EST + EPLBD tend to result in a persistent and comparable loss of SO function, which is a likely cause of duodenobiliary reflux, bacterial contamination of the biliary tract, and subsequent late adverse events, including stone recurrence, cholangitis, or liver abscess [22]. Finally, the greatest concern following EST is the likelihood of developing subsequent cholangiocarcinoma, and a number of authors have suggested that we be prudent to avoid a sphincterotomy in the very young until further studies clarify the situation in these patients [23]. Although both procedures are equivalent in terms of clinical outcomes, the single-stage surgical strategy can preserve the function of the SO, which effectively eliminates the potential serious risks of ERCP-associated complications and the need for further procedures [20, 24]. Moreover, the single-stage surgical strategy has a similar or better stone clearance rate, and it may be an ideal method for patients who fail ERCP because of difficult choledocholithiasis [25]. The single-stage surgical strategy has also been found to be safe and efficacious even in elderly patients [26]. Therefore, a single-stage surgical strategy is feasible and cost-effective, with a reduced length of hospital stay, and should ultimately be the preferred procedure for most patients [20, 24].

In the early stages of laparoscopy, previous abdominal surgery was widely regarded as a relative contraindication of laparoscopic surgery for the following reasons: (1) serious adhesions near the previous incision at the inner side of the abdominal wall make it possible to injure adjacent organs or tissues when inserting the first trocar or Veress needle; (2) it is difficult to distinguish and dissect extensive dense adhesions around the hepatoduodenal ligament and the CBD, which may increase the risk of injury to biliary and vascular structures at the hilar area [27]; and (3) these complex operations can be even further magnified by laparoscopy considering the lack of manual palpation and may ultimately increase the probability of conversion to laparotomy or even become a potential source of morbidity and prolonged hospital stay [27]. However, with the developments in laparoscopic techniques and technological advances in equipment, more surgeons have attempted to perform complicated laparoscopic surgeries in patients with previous abdominal surgery [28, 29]. Unfortunately, owing to the dense adhesions in the operative area, there are very few reports about laparoscopic procedures in patients with a history of abdominal biliary tract surgery. However, with the developments in laparoscopic equipment and the accumulation of experience by laparoscopic surgeons, complicated laparoscopic procedures can be performed in patients who have undergone previous biliary tract operations in our center. Based on our findings, we would object to the previous view that previous abdominal surgery is a relative contraindication for laparoscopic surgery.

In the present study, we found that the LCBDE group performed better than the OCBDE group with respect to estimated blood loss, remnant CBD stones, and postoperative hospital stay. First, the estimated blood loss of 30 (5–700) mL observed in the LCBDE group was similar to the estimated blood loss of 52 mL reported in Pang’s research [30]. The lower blood loss in the LCBDE group might be attributed to the mini-incision, clear vision, and delicate maneuvering. Second, the incidence of residual stones after LCBDE was reported to be 2% to 5% in retrospective studies of 157 patients and 170 patients, respectively [3, 31]. The present study showed that the proportion of residual stones following LCBDE was 17%, which was slightly higher than that previously reported. The patients in our study had a history of at least one abdominal biliary tract surgery, which significantly increased the difficulty of the operation in some patients. Additionally, some elderly and infirmed patients had a reduced tolerance to prolonged endoscopy surgery. Because the damage control strategy is recommended to reduce the incidence of adverse reactions resulting from prolonged endoscopy surgery, we preferred to address residual stones by choledochoscopy in the subsequent follow-up. The LCBDE group had a lower incidence of remnant CBD stones, which should be attributed to optical magnification and direct visualization. Third, a randomized prospective study by Rhodes et al. demonstrated that the median hospital stay was significantly lower in a one-stage LCBDE group than in a two-stage ERCP group (preoperative/postoperative ERCP and LC). According to a recent report, the mean postoperative hospital stay fluctuated from 2.8 ± 0.1 to 11.0 ± 6.1 days for LCBDE [3, 32]. In this study, the postoperative hospital stay was 7 (3–78) days, which compares favorably with other published studies of LCBDE. The short postoperative hospital stay in the LCBDE group might be attributed to the small incision and early time to oral intake due to the early recovery of bowel function. Fourth, a recent meta-analysis showed that the operative time was 119.5 min and the conversion rate was 9% in LCBDE, which were lower than the 170 (60–480) min and 17%, respectively, obtained in our study [18]. All the procedures (including laparotomy and laparoscopic surgery) were performed by an experienced surgeon. The main reason for the differences in the operative time and conversion rate is that a number of patients with abdominal adhesion were recruited in this study, which increased the values of these two results.

Pulmonary diseases such as pneumonia and pleural effusion seemed to be the most common postoperative complications, affecting up to 32% and 42% in the LCBDE and OCBDE groups, respectively. In the present study, 96% of patients with pulmonary disease recovered through aerosol inhalation or anti-infective therapy. Only 1 patient in the LCBDE group and 2 patients in the OCBDE group with pleural effusions recovered through ultrasound-guided local drainage. Moreover, postoperative complications such as bile leakage may become a major problem for patients who undergo LCBDE. In this study, bile leakage occurred in only 5% and 10% of patients in the LCBDE and OCBDE groups, respectively, and 82% of patients recovered through symptomatic and supportive treatment. Only 1 patient in the LCBDE group and 2 patients in the OCBDE group with bile leakage received ultrasound-guided local drainage. In addition, the incidences of wound infection and postoperative bleeding were quite low in our study and comparable to those reported by other authors [3, 33].

There is little evidence in the literature to evaluate the factors affecting the degree of postoperative adhesion in patients with previous abdominal biliary tract surgery. In this study, we assessed the feasibility and safety of LCBDE in patients following abdominal biliary tract surgery, and we found a significant difference in the previous number of operations, previous surgical approach and previous operation scope among classes zero and 1, 2 and 3 (*p ≤ *0.05). After further comparison between groups with respect to the previous operation scope, we found a significant difference between previous gallbladder surgery and previous bile duct surgery and previous gallbladder surgery and previous gallbladder + bile duct surgery between classes zero and 1, 2 and 3, which suggested that patients who received cholecystectomy alone had less adhesion than those who received biliary tract surgery (including bile duct or gallbladder + bile duct). The reason many surgeons prefer OCBDE after biliary surgery is adhesion; however, our research suggests that LCBDE should also be recommended for patients with a history of cholecystectomy, few previous operations (< 2 times), or a history of laparoscopic surgery. Previous biliary tract surgery (including bile duct or gallbladder + bile duct other than cholecystectomy alone) often involves more organs and tissues, which may significantly increase the risk of postoperative adhesion. It is not surprising that multiple previous operations (≥ 2 times) were risk factors for postoperative adhesion because multiple operations always increase tissue damage and local fibrosis formation. In addition, a comparative clinical study in 26 patients suggested that laparoscopic surgery resulted in less adhesion formation, either on the operative or at the trocar entry sites, than laparotomy [15].

Here, we summarize some key points of the surgical procedure performed during our operation. (1) A reasonable location for the first trocar port is absolutely vital and should be in the periumbilicus and far from the previous incision to prevent injuring adjacent organs or tissues. The classic open technique (Hasson technique) is highly recommended for patients with previous upper abdominal biliary tract operations and is apparently much safer than the classic closed technique (Verres needle). Once the pneumoperitoneum has been established, the second trocar could be placed below the right costal margin under direct vision. (2) The adhesions between the abdominal wall and the omental tissue, abdominal wall, and internal organs and adhesions between organs or structures are always serious for patients with previous abdominal biliary tract surgery. Therefore, whether intra-abdominal adhesions could be separated without unnecessary injury is directly related to the success or failure of the surgery. Laparoscopic adhesiolysis could be implemented well by blunt and sharp dissection with the use of scissors, an ultrasonic scalpel or the head of the aspirator. We generally separated the adhesion between the abdominal wall and the omentum or the antrum first, and then we carefully separated along the liver surface to restore the anatomical structure of the normal gastric pylorus, duodenum, and duodenal ligament. The duodenum is always an anatomical landmark in the separation of adhesions. Conversion to laparotomy should be considered immediately to prevent unnecessary damage to the clear tissue spaces that could not be accessed, or complications could occur during the laparoscopy. (3) Regarding the separation of the CBD, the ‘make a breakthrough at the center and dissect on the two sides’ technique should be employed, and the CBD should be identified clearly and dissected close to the visceral surface of the liver when the hepatoduodenal ligament and the Calot triangle are dissected. If it is difficult to distinguish the CBD, the surrounding anatomic marks, such as the cystic duct stump or duodenal bulb, may be helpful. It can also be further confirmed by bile duct puncture with a syringe at the same time, and the bile can be sent for culture. (4) The upper bile duct at the junction between the cystic duct and the common hepatic duct should be selected as the incision site. Moreover, the appropriate incision is suggested according to the diameter of the choledochoscope and the size and location of the stones. It would be better to use picture-in-picture dynamic display mode with laparoscopic and choledochoscopy dual vision in the process of stone extraction, which could help reduce the incidence of bile duct injury. (5) If the stones in the CBD are large, the stones should be removed by a variety of removal methods (crushed by laparoscopic grasping forceps or fragmented by electrohydraulic lithotripsy), and then the fragmentations could be extracted rather than being removed by expanding the incision to avoid damage to the wall of the bile duct. (6) Regarding the routine flexible choledochoscope examination, if a flexible choledochoscope is used to enter the dilated intrahepatic or extrahepatic duct, care should be taken to determine whether there are any problems, including bile duct stricture and tumor or residual stones in the bile duct, and the stones—including those of the dilated bile duct—should be removed if necessary. (7) To prevent biliary fistula and promote healing of the CBD incision, the CBD should be full-thickness sutured with a 4-0 absorbable single-layer suture.

There are also some potential limitations to the present study. This was a retrospective observational study, and some key statistics could not be measured, and significant biases might have affected the selection of controls. This was minimized by analyzing a consecutive series of patients who had undergone surgery by a single surgeon. Although the outpatient follow-up period in our study was more than six months, the study still lacked long-term follow-up data to investigate long-term complications. We will overcome these shortcomings in future research.

In summary, LCBDE is a safe and effective technology for the treatment of CBD stones in patients with a history of abdominal biliary tract surgery; it is minimally invasive and has less estimated blood loss, a lower incidence of remnant CBD stones, a shorter postoperative hospital stay, and fewer complications than other CBD procedures. LCBDE should be recommended for patients with a history of cholecystectomy, few previous operations (< 2 times), or a history of laparoscopic surgery. The technology is safe, effective, and promising for clinical application as long as its indications are taken seriously and skilled laparoscopic and choledochoscopy techniques are available.

## References

[1] Manes G, Paspatis G, Aabakken L, Anderloni A, Arvanitakis M, Ah-Soune P, Barthet M, Domagk D, Dumonceau JM, Gigot JF, Hritz I, Karamanolis G, Laghi A, Mariani A, Paraskeva K, Pohl J, Ponchon T, Swahn F, Ter Steege R, Tringali A, Vezakis A, Williams EJ, van Hooft JE (2019). Endoscopic management of common bile duct stones: European society of gastrointestinal endoscopy (ESGE) guideline. Endoscopy.

[2] Targarona EM, Bendahan GE (2004). Management of common bile duct stones: controversies and future perspectives. HPB.

[3] Lee HM, Min SK, Lee HK (2014). Long-term results of laparoscopic common bile duct exploration by choledochotomy for choledocholithiasis: 15-year experience from a single center. Ann Surg Treat Res.

[4] Gad EH, Zakaria H, Kamel Y, Alsebaey A, Zakareya T, Abbasy M, Mohamed A, Nada A, Abdelsamee MA, Housseni M (2019). Surgical (open and laparoscopic) management of large difficult CBD stones after different sessions of endoscopic failure: a retrospective cohort study. Ann Med Surg.

[5] Goong HJ, Moon JH, Lee YN, Choi HJ, Choi S, Choi MH, Kim MJ, Lee TH, Park S, Lee HK (2017). The role of endoscopic biliary drainage without sphincterotomy in gallstone patients with cholangitis and suspected common bile duct stones not detected by cholangiogram or intraductal ultrasonography. Gut Liver.

[6] Ballou J, Wang Y, Schreiber M, Kiraly L (2019). 10 years of laparoscopic common bile duct exploration: a single tertiary institution experience. Am J Surg.

[7] Choibased HR, Kim JD, Choi DL (2016). Laparoscopic common bile duct exploration: a feasible option for choledocholithiasis in patients with previous gastrectomy. J Minim Invasive Surg.

[8] Lyu Y, Cheng Y, Li T, Cheng B, Jin X (2018). Laparoscopic common bile duct exploration plus cholecystectomy versus endoscopic retrograde cholangiopancreatography plus laparoscopic cholecystectomy for cholecystocholedocholithiasis: a meta-analysis. Surg Endosc.

[9] Yun KW, Ahn YJ, Lee HW, Jung IM, Chung JK, Heo SC, Hwang K, Ahn HS (2012). Laparoscopic common bile duct exploration in patients with previous upper abdominal operations. Korean J Hepato-Biliary-Pancreat Surg.

[10] Wang Y, Bo X, Wang Y, Li M, Shen S, Suo T, Pan H, Liu H, Liu H (2017). Laparoscopic surgery for choledocholithiasis concomitant with calculus of the left intrahepatic duct or abdominal adhesions. Surg Endosc.

[11] Karayiannakis AJ, Polychronidis A, Perente S, Botaitis S, Simopoulos C (2004). Laparoscopic cholecystectomy in patients with previous upper or lower abdominal surgery. Surg Endosc.

[12] Luciano AA, Hauser KS, Benda J (1983). Evaluation of commonly used adjuvants in the prevention of postoperative adhesions. Am J Obstet Gynecol.

[13] Operative Laparoscopy Study Group (1991). Postoperative adhesion development after operative laparoscopy: evaluation at early second-look procedures. Fertil Steril.

[14] Skube SJ, Hu Z, Arsoniadis EG, Simon GJ, Wick EC, Ko CY, Melton GB (2017). Characterizing surgical site infection signals in clinical notes. Stud Health Technol Inform.

[15] Polymeneas G, Theodosopoulos T, Stamatiadis A, Kourias E (2001). A comparative study of postoperative adhesion formation after laparoscopic vs open cholecystectomy. Surg Endosc.

[16] Chapron C, Guibert J, Fauconnier A, Vieira M, Dubuisson JB (2001). Adhesion formation after laparoscopic resection of uterosacral ligaments in women with endometriosis. J Am Assoc Gynecol Laparosc.

[17] Bhandari TR, Shahi S, Bhandari R, Poudel R (2017). Laparoscopic cholecystectomy in the elderly: an experience at a tertiary care hospital in western nepal. Surg Res Pract.

[18] Gao YC, Chen J, Qin Q, Chen H, Wang W, Zhao J, Miao F, Shi X (2017). Efficacy and safety of laparoscopic bile duct exploration versus endoscopic sphincterotomy for concomitant gallstones and common bile duct stones: a meta-analysis of randomized controlled trials. Medicine.

[19] Minakari M, Samani RR, Shavakhi A, Jafari A, Alijanian N, Hajalikhani M (2013). Endoscopic papillary balloon dilatation in comparison with endoscopic sphincterotomy for the treatment of large common bile duct stone. Adv Biomed Res.

[20] Kenny R, Richardson J, McGlone ER, Reddy M, Khan OA (2014). Laparoscopic common bile duct exploration versus pre or post-operative ERCP for common bile duct stones in patients undergoing cholecystectomy: is there any difference?. Int J Surg.

[21] Szary NM, Al-Kawas FH (2013). Complications of endoscopic retrograde cholangiopancreatography: how to avoid and manage them. Gastroenterol Hepatol.

[22] Cheon YK, Lee TY, Kim SN, Shim CS (2017). Impact of endoscopic papillary large-balloon dilation on sphincter of Oddi function: a prospective randomized study. Gastrointest Endosc.

[23] Oliveira-Cunha M, Dennison AR, Garcea G (2016). Late complications after endoscopic sphincterotomy. Surg Laparosc Endosc Percutan Tech.

[24] Rogers SJ, Cello JP, Horn JK, Siperstein AE, Schecter WP, Campbell AR, Mackersie RC, Rodas A, Kreuwel HT, Harris HW (2010). Prospective randomized trial of LC + LCBDE vs ERCP/S + LC for common bile duct stone disease. Arch Surg.

[25] Gupta N (2016). Role of laparoscopic common bile duct exploration in the management of choledocholithiasis. World J Gastrointest Surg.

[26] Zheng C, Huang Y, Xie E, Xie D, Peng Y, Wang X (2017). Laparoscopic common bile duct exploration: a safe and definitive treatment for elderly patients. Surg Endosc.

[27] Cipriani F, Ratti F, Fiorentini G, Catena M, Paganelli M, Aldrighetti L (2018). Effect of previous abdominal surgery on laparoscopic liver resection: analysis of feasibility and risk factors for conversion. J Laparoendosc Adv Surg Tech A.

[28] Akyurek N, Salman B, Irkorucu O, Tascilar O, Yuksel O, Sare M, Tatlicioglu E (2005). Laparoscopic cholecystectomy in patients with previous abdominal surgery. JSLS.

[29] Yamamoto M, Okuda J, Tanaka K, Kondo K, Asai K, Kayano H, Masubuchi S, Uchiyama K (2013). Effect of previous abdominal surgery on outcomes following laparoscopic colorectal surgery. Dis Colon Rectum.

[30] Pang L, Zhang Y, Wang Y, Kong J (2018). Transcystic versus traditional laparoscopic common bile duct exploration: its advantages and a meta-analysis. Surg Endosc.

[31] Quaresima S, Balla A, Guerrieri M, Campagnacci R, Lezoche E, Paganini AM (2017). A 23 year experience with laparoscopic common bile duct exploration. HPB.

[32] Riciardi R, Islam S, Canete JJ, Arcand PL, Stoker ME (2003). Effectiveness and long-term results of laparoscopic common bile duct exploration. Surg Endosc.

[33] Chan DSY, Jain PA, Khalifa A, Hughes R, Baker AL (2014). Laparoscopic common bile duct exploration. Brit J Surg.

